# Restoring anticipatory control through cognitive–motor dual-task training improves emotional and executive function in frail older adults

**DOI:** 10.3389/fnagi.2026.1890281

**Published:** 2026-08-14

**Authors:** Luca Boccacci, Martina Scalia, Riccardo Borzuola, Valentina Camomilla, Domenico Campagna, Andrea Casella, Imma Ceriello, Natalie Ferrulli, Margherita Filosa, Chiara Fossati, Federica Galli, Andrea Macaluso, William Manzon, Camilla Panacci, Martina Parrella, Fabio Pigozzi, Sabrina Pitzalis, Franco Pio Siciliano, Arnaldo Zelli, Francesco Di Russo

**Affiliations:** 1 Department of Movement, Human and Health Sciences, University of Rome “Foro Italico”, Rome, Italy; 2 Santa Lucia Foundation IRCCS, Rome, Italy; 3 Centre for Exercise Science and Sports Medicine, Foro Italico University Foundation, Rome, Italy

**Keywords:** aging, anticipatory control, anxiety, bilateral alternated stimulation (BAS), cognitive–motor dual-task training, depression, event-related potentials (ERP), frailty

## Abstract

**Background:**

Aging-related frailty is increasingly recognized as a multidimensional condition involving impairments in cognitive, emotional, and motor domains. Traditional interventions often fail to capture the complexity of real-world functioning, highlighting the need for more ecologically valid approaches.

**Objectives:**

This study investigated the effects of a structured Cognitive–Motor Dual-Task Training (CMDT) protocol, incorporating alternating bilateral stimulation (BAS), on emotional regulation, executive function, and anticipatory neural mechanisms in frail and pre-frail older adults.

**Methods:**

Thirty participants aged ≥ 65 years were enrolled in a randomized controlled trial and assigned to either a CMDT group or a motor-only control group. The intervention lasted 8 weeks (two sessions per week). Assessments were conducted at baseline, mid-intervention, and post-intervention, and included measures of anxiety (STAI-Y), depression (BDI-II), executive performance (reaction times, variability, and accuracy), functional mobility (BBS, TUG, 10mWT), and high-density EEG during a Go/No-go task to extract anticipatory event-related potentials (prefrontal negativity, pN; Bereitschaftspotential, BP).

**Results:**

The CMDT group showed significant reductions in anxiety and depressive symptoms, along with improved executive performance compared to the control group. At the neural level, CMDT enhanced anticipatory activity, reflected by increased pN and BP amplitudes. Mediation analyses revealed that changes in prefrontal anticipatory activity (pN) significantly mediated the relationship between CMDT and emotional improvements, with a full mediation pattern observed for self-reported depressive symptoms and a partial mediation pattern for anxiety.

**Conclusion:**

These findings indicate that CMDT enhances anticipatory control mechanisms linking brain function to behavior, supporting both emotional regulation and executive efficiency in frail older adults. By embedding cognitive demands within motor actions, this approach provides a low-cost and scalable intervention with increased ecological relevance, offering a promising strategy for addressing multidimensional vulnerability in aging.

## 1 Introduction

Population aging represents a rapidly expanding global phenomenon driven by increased life expectancy and declining fertility rates (Beard et al., 2016). This demographic shift has profound socio-economic consequences, imposing increasing demands on healthcare and pension systems, which already account for a substantial proportion of public expenditure in advanced economies. It is estimated that by 2050, approximately 2 billion individuals worldwide will be over the age of 60, with nearly one quarter of the global burden of disease concentrated in this age group (Harper, 2014; Prince et al., 2015). Within this context, frailty has emerged as a critical condition associated with aging. Frailty is a multidimensional clinical syndrome characterized by reduced physiological reserve and increased vulnerability to stressors, resulting from cumulative declines across multiple biological systems and associated with adverse outcomes including disability, falls, hospitalization, and mortality (Clegg et al., 2013; Fried et al., 2001; Rockwood and Mitnitski, 2007). Recent evidence indicates that, in frail older adults, reduced prefrontal cortex activity is associated with elevated anxiety and impaired cognitive control (Boccacci et al., 2026), highlighting the central role of frontal networks in emotional and executive dysfunction within this population. Importantly, frailty is increasingly conceptualized not only as a physical condition, but as a state of reduced adaptive capacity within distributed control systems, particularly those subserved by the prefrontal cortex. This vulnerability affects the ability to anticipate, plan, and regulate action and emotion, rendering frail older adults especially susceptible to anxiety, depression, and executive inefficiency (e.g., Bartoli et al., 2020; Pozo et al., 2023; Soysal et al., 2017; Tan et al., 2023). Within this framework, interventions that directly target anticipatory and proactive control mechanisms may exert particularly strong effects on emotional regulation.

One of the methods used to contrast the physical and cognitive impairment associated with frailty in older adults is Cognitive-Motor Dual-Task (CMDT) training (e.g., Lai et al., 2025). CMDT refers to the simultaneous performance of a motor task (e.g., walking, balance, coordinated movements) and a concurrent cognitive task (e.g., executive control, attention, working memory, decision-making). During CMDT, motor and cognitive processes interact and compete for shared neural resources, particularly within prefrontal and fronto-parietal control networks. Performance under CMDT conditions is sensitive to attentional load, executive demand, and task complexity, making it a powerful tool to mitigate the physical and cognitive decline of older adults (e.g., Fritz et al., 2015; Tao et al., 2022; Tuena et al., 2023).

From a neurophysiological perspective, CMDT engages distributed brain networks, including the prefrontal cortex, premotor and motor areas, basal ganglia, and parietal regions, and has been shown to modulate preparatory and task-related neural activity indexed by EEG, ERP, and other functional measures (e.g., Tuena et al., 2023). Models of neuroplasticity and compensation suggest that CMDT training induces a functional reorganization of neural networks, with particular involvement of the prefrontal areas (Cabeza, 2002; Park and Reuter-Lorenz, 2009). In this context, dual-tasking is initially associated with an increase in cognitive-motor interference, followed, with repeated exposure, by a gradual adaptation and greater efficiency in the allocation of attentional resources (Woollacott and Shumway-Cook, 2002; Pashler, 1994). The meta-analysis by Wollesen et al. (2020), comprising 25 studies conducted on older adults, highlights that CMDT training interventions result in significant improvements in global cognition and inhibitory control, as well as beneficial effects on attention, processing speed, and performance in dual-task conditions.

The literature has highlighted the use of various types of CMDT treatment aimed at enhancing cognitive-motor performance in older adults, such as the CMDT used by Lucia et al. (2024), in which electronic devices were used to allow participants to interact with cognitive stimuli via motion sensors, incorporating an explicit and structured integration of cognitive and motor tasks. This training not only improved physical and cognitive performance but also reduced anxiety levels. These findings suggest that anxiety symptoms may be modulated through interventions targeting anticipatory control mechanisms, without necessarily relying on psychotherapeutic or pharmacological approaches, positioning CMDT as a mechanism-driven intervention acting on distributed prefrontal–premotor networks. Besides anxiety, another condition with high prevalence and substantial socioeconomic impact is depression. According to the World Health Organization (World Health Organization [WHO], 2025), depression affects approximately 280 million people worldwide and is among the leading causes of disability globally, with mental disorders affecting around 14% of adults aged 60 years and older. Both conditions share dysfunctions in frontoparietal circuits and cognitive and emotional control mechanisms (e.g., Williams, 2016), paving the way for combined and personalized interventions. CMDT training has been found to moderately reduce depression, but with contrasting results (e.g., Nascimento et al., 2022; Schoene et al., 2014, 2015; Ye et al., 2024). This heterogeneity may reflect the fact that depressive symptoms are influenced by multiple interacting cognitive, affective, social, and neurobiological mechanisms. In this context, CMDT-related improvements in self-reported depressive symptoms may be partly associated with enhanced prefrontal engagement and goal-directed control; however, the present evidence does not allow definitive conclusions regarding whether these effects are direct, indirect, or mediated by multiple interacting pathways. Direct evidence that CMDT produces stronger and more reliable effects on anxiety than on depression could be due to anxiety’s tighter link with anticipatory control (e.g., Lucia et al., 2024). Transdiagnostic circuit models show that anxiety is more directly coupled to control-network dysregulation than depression, which may account for differential responsiveness (Williams, 2016; Tozzi et al., 2024).

To better target depression, we sought to exploit a method known as Bilateral Alternating Stimulation (BAS), which was introduced within the framework of Eye Movement Desensitization and Reprocessing (EMDR) psychotherapy (Shapiro, 1989) as a central component of a protocol designed to facilitate the processing of traumatic memories. Subsequently, this procedure was incorporated into the Adaptive Information Processing (AIP) model, according to which bilateral stimulation promotes the integration of dysfunctional information within adaptive memory networks (Shapiro, 2001). Various theoretical interpretations have been proposed to explain the underlying mechanisms, including the model of interhemispheric interaction, which suggests an enhancement of communication between the cerebral hemispheres (Christman et al., 2003). Furthermore, alternating bilateral stimulation modulates interhemispheric coherence, potentially favoring a reorganization of communication processes between hemispheres that could facilitate the integration of cognitive and emotional information (Propper et al., 2007). It also modulates prefrontal cortex activity and fronto-limbic connectivity, supporting a reorganization of cognitive and emotional control processes (Pagani et al., 2012, 2015; Herkt et al., 2014). More recent electroencephalographic (EEG) evidence confirms the involvement of frontal areas during BAS in line with theoretical models that attribute these effects to the modulation of attentional and executive systems (Stingl et al., 2025; Coubard, 2016).

Among anticipatory EEG markers, the prefrontal negativity (pN) and the Bereitschaftspotential (BP) are particularly relevant for investigating the neurophysiological mechanisms underlying cognitive-motor interventions. The pN is considered an index of proactive cognitive control and top-down anticipatory processes, reflecting the allocation of executive resources before stimulus onset (Di Russo et al., 2019). In contrast, the BP reflects premotor preparation and motor planning preceding voluntary movement (Shibasaki and Hallett, 2006). Previous work has shown that frail older adults exhibit reduced anticipatory prefrontal activity, suggesting impaired proactive cognitive control (Boccacci et al., 2026). Based on this evidence, we hypothesized that CMDT would primarily increase pN amplitude, reflecting improved anticipatory cognitive control, and secondarily enhance BP amplitude, indicating more efficient motor preparation.

In light of the existing literature and relevant theoretical models, this study aimed to evaluate the effectiveness of a CMDT training intervention based on structured motor protocols and the use of BAS during the motor activity. It has been hypothesized that this intervention could enhance the attentional and executive systems and improve motor performance in participants. Furthermore, it was anticipated that the training could produce a reduction in levels of anxiety and depress ion in the frail elderly population aged over 65. At the neurophysiological level, we further hypothesized that CMDT would primarily increase prefrontal negativity (pN) amplitude, reflecting enhanced anticipatory cognitive control, and secondarily enhance Bereitschaftspotential (BP) amplitude, indicating more efficient motor preparation. Accordingly, these neurophysiological changes were expected to accompany improvements in behavioral performance and emotional outcomes, with pN showing the strongest associations because of its established role in proactive executive control.

## 2 Materials and methods

### 2.1 Participants

A priori power analysis was conducted using R (version 4.5.3) and the PWR package. Since the main hypothesis focused on detecting a Group × Time interaction in a mixed-design ANOVA (two groups, three repeated measurements), standard analytical solutions do not accurately estimate power; we performed a simulation-based power analysis using multivariate normal simulations (5,000 iterations) with empirical effect sizes observed in a previous CMDT randomized trial in older adults employing similar motor, cognitive, and neurophysiological outcomes (Aydin et al., 2026). In that study, Group × Time interaction effects ranged from Cohen’s f = 0.32–1.06, indicating medium-to-large effects. To avoid overestimating statistical power and to provide a conservative sample size estimate, we intentionally selected the smallest empirical Group × Time interaction effect (*f* = 0.32). Correlation between repeated measures was set to *r* = 0.50, σ = 5; the simulation indicated that a total sample size of 30 participants yields ≥ 0.80 power to detect a significant Group × Time interaction.

Participants were recruited from local senior centers and underwent an initial clinical screening conducted by experienced physicians to evaluate their general health status, pharmacological history, and the presence of a frail or pre-frail phenotype. Participation in the study was voluntary. All individuals provided written informed consent before participation, and they were not informed about the specific aims of the experimental procedures. The study was conducted in accordance with the ethical principles outlined in the World Medical Association’s Declaration of Helsinki and received approval from the research ethics committee of the University of Rome “Foro Italico” (approval #47-23, date 15/11/2023).

Eligibility criteria were: age ≥ 65 years; absence of dementia (Mini-Mental State Examination > 25; Folstein et al., 1975); no active neurological or diagnosed psychiatric disorders requiring current pharmacological or psychotherapeutic treatments; no substance abuse in the previous 6 months; no severe visual impairments; preserved functional autonomy (Activities of Daily Living, ADL ≥ 5; Katz et al., 1963); no impairment in Instrumental Activities of Daily Living (IADL ≥ 5; Lawton and Brody, 1969). Frailty was assessed using the Fried Frailty Phenotype (Fried et al., 2001), which evaluates five criteria: unintentional weight loss, exhaustion, low physical activity, slow gait speed, and weakness. Each criterion was coded dichotomously (0/1), and the total score was used to classify individuals as robust (0–2), pre-frail (3), or frail (4–5). Frail and pre-frail individuals were included in the study. After applying this screening on 94 potential community-dwelling older adults, 30 were recruited for the study (22 females and 8 males, mean age 78.9 ± 5.2 years). Eligibility and clinical status were assessed by experienced geriatricians, who performed the clinical evaluation and verified all inclusion and exclusion criteria prior to enrollment.

### 2.2 Study design and procedure

The designed protocol included three training arms: cognitive–motor training, motor training with core-specific exercises, and general motor training. Participants were randomly allocated to three groups using a computer-generated random sequence. Before data analysis, and according to preregistered methodological criteria (ClinicalTrials.gov Identifier: NCT07546734), participants who performed motor training with core-specific exercise and general motor training were merged into a single control group. This decision was justified by their functional equivalence and by the need to optimize statistical power and interpretability when comparing cognitive-motor training with motor-only exercise. Preliminary analyses confirmed that the two motor control groups did not differ significantly in baseline characteristics or outcome trajectories. The unification of the control groups allowed isolating the specific contribution of the cognitive component embedded within the experimental intervention. Accordingly, the final sample included a CMDT experimental group comprising 10 participants (mean age 77.8 ± 3.4 years; nine females) and a control motor group comprising 20 participants (mean age 79.9 ± 5.3 years; 13 females). Handedness was assessed using the Edinburgh Handedness Inventory, and all participants were right-handed. Preliminary analyses confirmed that the groups did not significantly differ in terms of sex, age, educational level, or socioeconomic status (X^2^ and *t*-test; were not significant).

**FIGURE 1:**
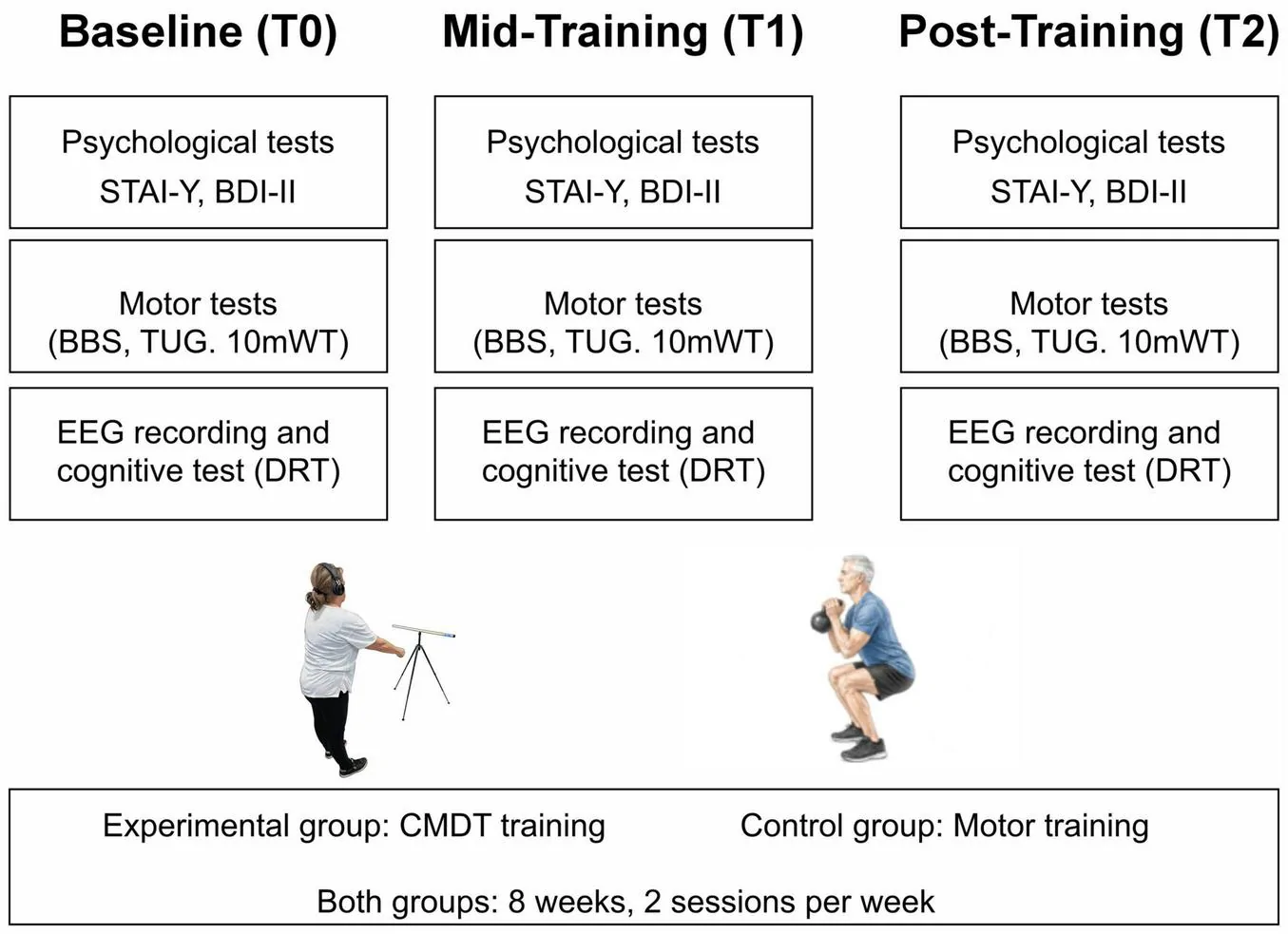
Schematic representation of the experimental procedure. Participants underwent three assessment sessions: baseline (T0), mid-training (T1, after 4 weeks), and post-training (T2, after 8 weeks). At each time point, psychological assessments were administered to evaluate anxiety (STAI-Y) and depression (BDI-II). Motor tests assessed physical performance (BBS, TUG, and 10mWT). In addition, electrophysiological activity was recorded during a visuomotor discrimination response task (DRT) performed under EEG monitoring to assess anticipatory cognitive and motor processes. Between assessments, participants completed an 8-week intervention consisting of either cognitive–motor dual-task CMDT training (experimental group) or motor training alone (control group), with two sessions per week. The right illustration generated using Microsoft 365 Copilot from text prompts provided by the authors (see Supplementary material).

All groups participated in an 8-week training program with sessions conducted twice per week for one hour. The control group sessions entailed exclusively motor training activities. The experimental group followed the same motor training schedule, but the exercises were implemented using the CMDT protocol described below. Assessments were conducted at three time points: before the intervention (T0), after 4 weeks (T1) of training, and immediately following the completion of the training program (T2). At each assessment, participants completed a battery of psychological measures and motor tests (as detailed in Figure 1), in addition to a cognitive task performed while electroencephalographic (EEG) activity was recorded. T0 and T2 assessments were administered 1–2 days before and after the treatment. A schematic representation of the experimental procedure is reported in Figure 1. Since the project terminated after T2 completion, no retention tests could be administered; therefore, no data regarding the persistence of training effects could be collected.

Importantly, training volume, physical workload, instructor supervision, and social interaction were comparable across control and experimental groups. Training dose and structure were standardized across participants by maintaining the same number of sets, repetitions, session duration, exercise progression, and therapist supervision throughout the intervention. However, objective physiological indicators of exercise intensity, such as heart rate or ratings of perceived exertion, were not collected. Therefore, any between-group differences observed in cognitive, emotional, or neurophysiological outcomes can be attributed to the presence of concurrent cognitive demands and anticipatory control requirements in the CMDT protocol rather than to differences in physical exercise exposure.

### 2.3 Emotion tests

Anxiety levels were assessed using the State–Trait Anxiety Inventory, Form Y (STAI–Y, Spielberger et al., 1983), widely considered a standard instrument for the assessment of anxiety. The STAI consists of two self-report subscales designed to measure state anxiety and trait anxiety. State anxiety (STAI–Y1) refers to a transient emotional condition characterized by feelings of tension, apprehension, and heightened autonomic activity. In contrast, trait anxiety (STAI–Y2) reflects a relatively stable personality disposition associated with a tendency to perceive situations as threatening and to respond with persistent worry, agitation, and nervousness. Total scores for each subscale range from 20 to 80, with commonly adopted interpretative thresholds indicating low or absent anxiety (20–37), moderate anxiety (38–44), and high anxiety (45–80).

Depression levels were assessed using the Beck Depression Inventory-II (BDI-II, Beck et al., 1996), which is a self-report instrument used to assess the severity of depressive symptoms in adolescents and adults. The questionnaire consists of 21 items that investigate cognitive, affective, and somatic symptoms of depression. Each item is rated on a scale of 0–3, with reference to the last 2 weeks. The total score, obtained from the sum of the responses (range 0–63), reflects the severity of depressive symptoms. According to standard interpretative guidelines, scores are classified as no or minimal (0–13) depression, mild (14–19), moderate (20–28), and severe (29–63) depression. Participants completed the questionnaires independently. Both instruments are well-validated self-report measures widely used to assess depressive and anxiety symptoms. Accordingly, the present findings reflect participants’ subjective reports of emotional symptoms rather than clinician-rated diagnoses and should be interpreted within this methodological context.

### 2.4 Motor tests

Balance was assessed using the Berg Balance Scale (BBS, Berg et al., 1992), which is a standardized clinical tool used to assess static and dynamic balance in older adults. It consists of 14 functional tasks representative of everyday activities; each is scored on a 5-point scale (0–4). Total scores range from 0 to 56, with higher scores indicating better balance and lower fall risk (41–56: low fall risk, 21–40: medium fall risk, 0–20: high fall risk). The BBS is widely used in clinical and research settings due to its high reliability and validity in aging populations. The BBS was administered according to standardized procedures by trained evaluators.

Functional mobility was assessed using the Timed Up and Go (TUG, Podsiadlo and Richardson, 1991) test, which is a standardized test used to assess functional mobility, balance, and fall risk in older adults. Participants are instructed to stand up from a standard chair, walk 3 m at a comfortable pace, turn around, walk back to the chair, and sit down. The total time required to complete the task (completion time) is recorded in seconds using a stopwatch, with shorter times indicating better functional mobility. The TUG is a reliable and valid measure widely used in both clinical and research settings.

Walking performance was assessed using the 10-Meter Walking Test (10mWT, Wolf et al., 1999). Establishing the reliability and validity of measurements of walking time using the Emory Functional Ambulation Profile. Physical Therapy, 79(12), 1,122–1,133), administered at both comfortable speed (10mWt_CS) and fast speed (10mWt_FS). Participants were instructed to walk along a straight walkway and were timed over a central 10-m distance, with acceleration and deceleration phases excluded from analysis. For the comfortable speed condition, participants were asked to walk at their usual, self-selected pace. For the fast speed condition, they were instructed to walk as fast as possible while maintaining safety and without running. Walking speed was calculated in meters per second (m/s) by dividing the distance by the time required to complete the 10-m section. Both measures provide reliable and valid indices of functional mobility and gait capacity in older adults, with comfortable speed reflecting habitual walking behavior and fast speed indexing maximal locomotor reserve.

### 2.5 Cognitive test

A visuomotor discrimination response task (DRT), based on a Go/No-go paradigm, was included to assess executive control processes involved in task anticipation, including both cognitive and motor preparation, which are known to be particularly sensitive to aging and to cognitive-motor training effects. This task is particularly well-suited for electrophysiological investigation, as it reliably elicits pre-stimulus anticipatory ERP components, such as the Bereitschaftspotential (BP) and the prefrontal negativity (pN). These components are associated with motor preparation and top-down cognitive control, respectively, allowing the assessment of predictive brain activity that precedes action execution. For this reason, during the DRT, high-density EEG was also recorded (see dedicated section).

**FIGURE 2:**
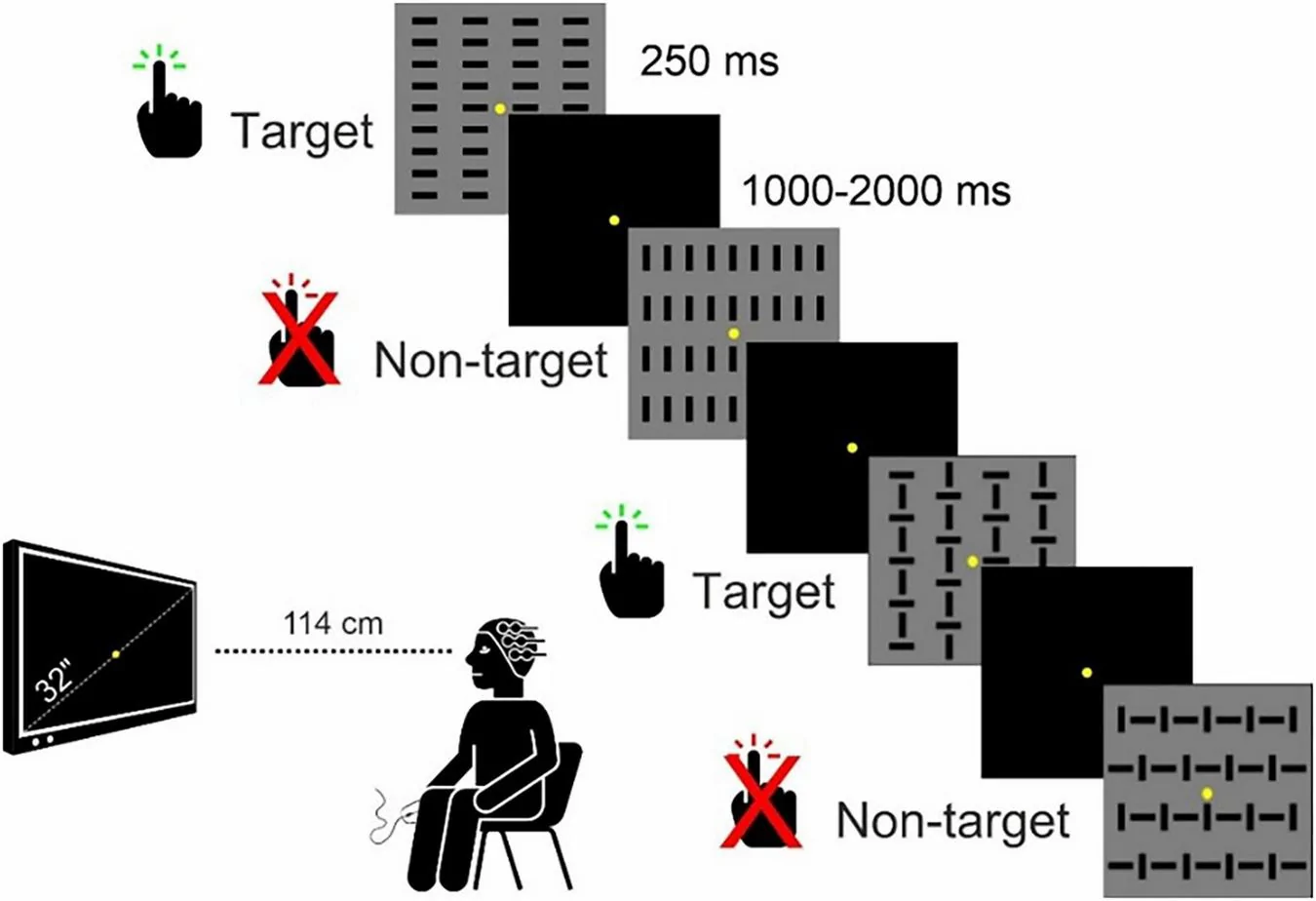
Schematic representation of the Go/No-Go discrimination response task (DRT). Each trial began with a fixation point presented on a black background for a variable interval (1–2 s), followed by the brief presentation (250 ms) of one of four visual stimuli. Two stimuli were designated as targets, requiring a speeded button press with the right index finger, whereas responses had to be withheld for non-target stimuli. Target and non-target trials occurred with equal probability.

After the EEG cap was applied to the scalp, participants were examined in a dimly lit, sound-attenuated room. With their right-hand palm down on a push-button board, they were sitting in front of a computer screen that was 114 cm away from their eyes. Throughout the whole experiment, a white fixation point (diameter circle 0.15 × 0.15) on a black backdrop remained in the middle of the screen. Four visual stimuli were randomly presented for 250 ms with equal probability (*p* = 0.25); the stimulus–onset asynchrony ranged from 1 to 2 s to avoid ERP overlaps with prior and subsequent stimuli and stimulus prediction. Only when two of the four predefined target stimuli appeared on the screen (*p* = 0.5) did participants need to click the button with their right index finger as quickly as feasible and to suppress the motor response when non-target stimuli emerged (*p* = 0.5); the investigator placed equal emphasis on speed and accuracy. The stimuli and paradigm used in the task are schematically represented in Figure 2. Between runs, the four stimuli were presented in a different sequence. Each run lasted two minutes, with a wait in between. Depending on each person’s rest period during pauses, 10 runs were conducted, enabling us to gather 400 trials for each stimulus category in around 25–30 min.

### 2.6 EEG recording and ERP analysis

Two BrainAmp*™* amplifiers connected to ActiCap’s 64 active sensors were used to record a continuous electroencephalogram (EEG) during the cognitive test. The used software was Recorder 1.2 and Analyzer 2.3, both developed by BrainProducts GmbH in Gilching, Germany. The average of the M1-M2 electrodes was used to determine the reference. The scalp channels’ position was based on the 10-10 International System. The EEG signals were digitized at 250 Hz and band-pass filtered using a Butterworth zero-phase filter (0.01–80 Hz; second order) before being stored for offline analysis. The electrooculogram (EOG) was recorded using the third BrainAmp amplifier (ExG type), which tracked bipolar eye movements. Electrodes were placed above the outer canthi of the left eye to record the horizontal EOG, and above and below the left eye to capture the vertical EOG. The electrodes’ impedances were maintained below 10 KΩ for the active scalp electrode and 5 KΩ for EOG electrodes. The Analyzer’s independent component analysis tool was used to automatically detect the blink and vertical eye movement artifacts. Following that, EEG signals with amplitudes greater than the ± 70 μV threshold were eliminated from the data by automatically applying artifact rejection. Accordingly, about 7%–12% of trials were rejected, and these values did not differ between groups and conditions; consequently, averages were based on 700–750 trials. To measure pre-stimulus activity, EEG data were divided into 1,300 ms epochs that started 1,100 ms before the stimulus presentation and ended 200 ms later. The baseline was the first 200 ms (−1,100/−900 ms). Due to the unpredictability of the stimulus classification during the pre-stimulus interval, target and non-target trials were averaged.

**FIGURE 3:**
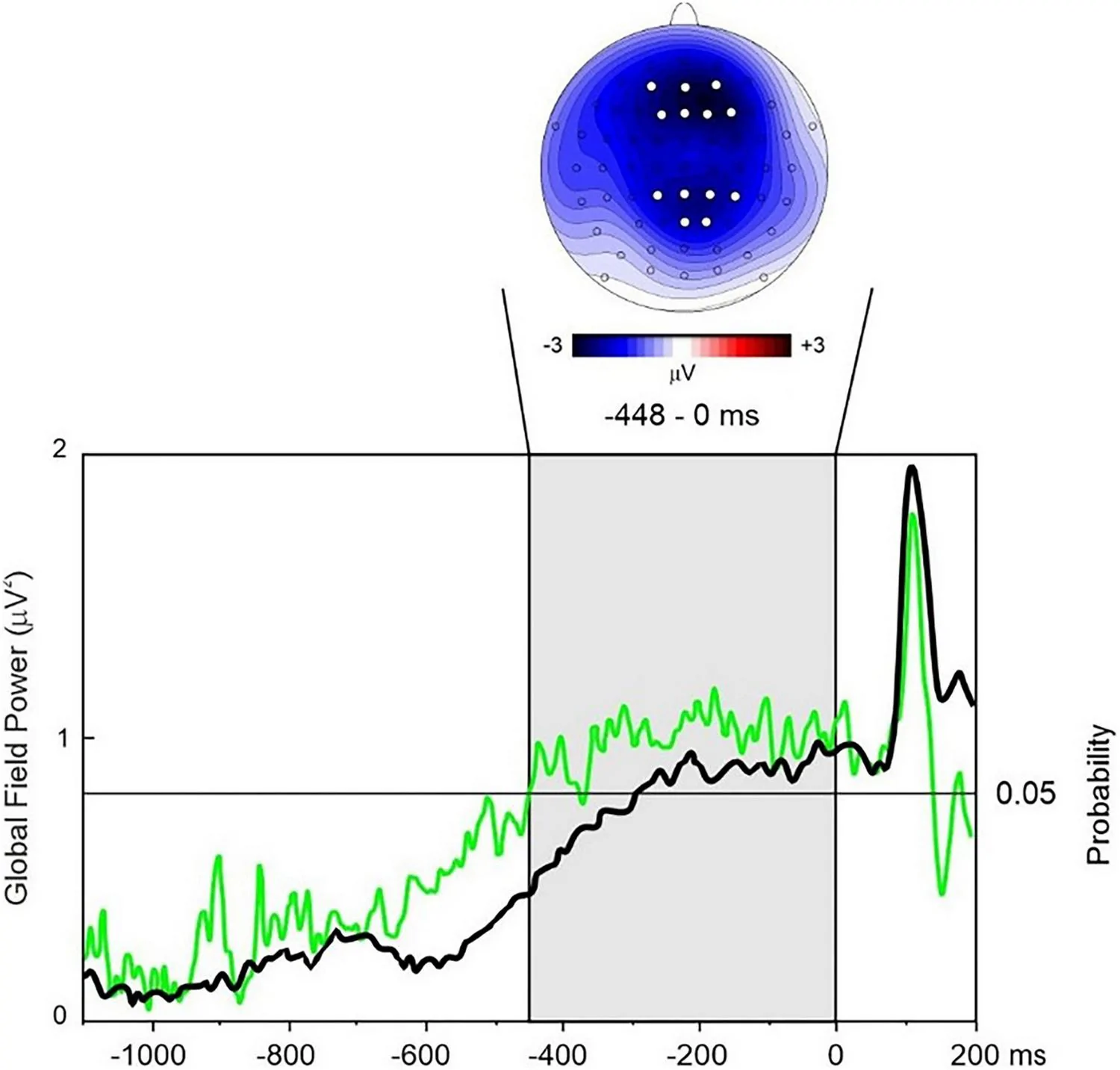
Global field power (GFP) of the collapsed localizers ERP (black lines), and the intervals selected based on the *t*-test against zero (green line corresponding to the *p*-value). Above the top-flat map in the –440 to 0 ms interval with the selected electrode highlighted.

The “collapsed localizer” technique (Luck and Gaspelin, 2017), which included averaging the ERP of all groups and circumstances to construct a localizer ERP, was used to define the intervals and electrodes for statistical analysis a-priori. By calculating the global field power (GFP) on each ERP and subsequently on the localizer, the analysis interval was ascertained. The GFP concurrently displays the spatial variability of the ERP across all scalp electrodes. Starting from time zero and working backward, the interval with significant values (*p* < 0.05) was chosen by running a *t*-test against zero on the GFP. For statistical analysis, the mean amplitude between −448 and 0 ms was computed in this way. A spatial pool was created by averaging the electrodes in the chosen localizer interval that had a *t*-test against zero with *p* < 0.05. A medial prefrontal pool (FP1, FPz, FP2, AF3, AFZ, AF4) representing the pN component and a medial centro-parietal pool (C1, Cz, C2, CP1, CPz, CP2) representing the BP component were found using this approach. Figure 3 shows the GPF and p time course and the scalp topography in the selected interval.

### 2.7 Motor training (control group)

The control intervention consisted exclusively of motor training programs designed to improve general physical fitness and functional mobility in older and frail adults, without the inclusion of explicit cognitive demands. In the original study design, participants assigned to the control condition were distributed into two distinct motor training groups, which differed only in the specific emphasis of the exercises but were equivalent in structure, intensity, and training volume.

The first control group (general motor training) followed a multicomponent exercise program targeting overall physical conditioning. Sessions included exercises focused on lower-limb strengthening, static and dynamic balance, joint mobility, and flexibility. Typical activities comprised standing and walking balance exercises, weight-bearing movements, controlled gait tasks, and gentle mobility drills. The second control group (core-focused motor training) followed a motor program emphasizing trunk stability and postural control. Exercises targeted core muscle activation, postural alignment, and coordination during standing and walking tasks, while maintaining the same overall level of physical effort and safety constraints as the general motor training group. Importantly, although core engagement was emphasized, training remained purely motor in nature and did not involve concurrent cognitive tasks, stimulus–response associations, or decision-making demands. Both control motor training protocols were administered twice per week for 60 min over 8 weeks, matching the experimental CMDT intervention in terms of session frequency, duration, total training volume, and group-based delivery. Exercises were conducted under the supervision of trained instructors, followed a structured progression across weeks, and were adapted as necessary to individual functional capacities to ensure safety and feasibility.

#### 2.7.1 Cognitive-motor training (experimental group)

Participants assigned to the experimental group completed a Cognitive–Motor Dual-Task Training (CMDT) program in which physical exercises were systematically combined with concurrent cognitive demands. The CMDT protocol was designed to simultaneously engage motor performance, executive functions, and attentional control, thereby enhancing the integration of cognitive and motor processes. The CMDT was embedded within the standard physical training session, replacing part of the motor-only exercises with structured cognitive–motor tasks, ensuring equivalence in physical workload and social interaction across groups.

The CMDT was implemented using the Witty-SEM™ system (Microgate Srl., Bolzano, Italy), a portable interactive training platform composed of LED-based devices equipped with proximity sensors. The devices displayed visual symbols (e.g., numbers, letters, arrows) in distinct colors and emitted auditory cues, prompting participants to execute specific motor actions (e.g., stepping, reaching, weight shifting) contingent on the presented signals. Responses were recorded in real time, enabling precise control of task demands. Task difficulty and cognitive load were progressively adjusted across sessions by manipulating stimulus complexity, response speed requirements, number of active devices, and rule-switching frequency. Initial task difficulty was calibrated individually, and progression followed predefined levels to maintain engagement while ensuring safety and feasibility for older and frail participants.

Sensory stimulations were delivered using a system (EMDR kit by SE Factory/EMDR kit Groningen, The Netherlands) that is a professional, portable set designed for psychotherapists to provide bilateral stimulation (BLS) to clients during EMDR therapy. The device was used to deliver alternating BLS through visual, tactile, or auditory stimuli presented alternately between the two sides of the body. The system includes a light bar with movable LEDs that guide eye movements and tactile stimulators (pulsators) that produce alternating vibrations in the participant’s hands. The headphones also stimulate in alternating bilateral mode through the presentation of a sound.

The CMDT program consisted of short exercise routines combining balance, locomotion, and strength activities with stimulus–response cognitive tasks targeting key executive functions, including attention, working memory, response inhibition, cognitive flexibility, and decision-making. Cognitive demands were intentionally integrated into movement execution, requiring participants to adapt their motor responses based on changing external cues or task rules. Specifically, participants were required to: respond selectively to target stimuli while inhibiting responses to non-target stimuli, perform movements according to predefined or changing stimulus–response associations, switch between different response rules, memorize and update short sequences of actions, reverse or randomize learned motor sequences to prevent habitual responding. The established executive function domains as described in the neuropsychological literature were intended to be engaged by these task components. In particular, rule switching involved cognitive flexibility, selective responding, and response inhibition focused on inhibitory control, and sequence memorizing, updating, and reversal mostly used working memory and updating processes. These executive domains are seen as essential elements of cognitive control and goal-directed behavior (Miyake et al., 2000; Diamond, 2013), and more recent research has consistently supported their applicability (Rodríguez-Nieto et al., 2022).

Importantly, unlike the control condition, the CMDT included explicit task goals and cognitive demands, fostering anticipatory processing, top-down control, and coordinated action planning. This design allowed the isolation of the specific contribution of the cognitive component embedded within physical training, enabling a direct comparison with motor training alone. Some exercises were made using alternating movements and sensory stimulation.

Participants were required to respond to lateralized stimuli by executing predefined motor actions, such as stepping, reaching, or shifting body weight toward the side indicated by the stimulus. The alternation of left–right cues required continuous updating of stimulus–response associations, increased demands on attentional allocation, response selection, and inhibition, and prevented the adoption of automatic or stereotyped movement patterns. The frequency and speed of bilateral stimulation were adjustable and were progressively modulated across training sessions to match individual capabilities and to maintain task challenge. Bilateral stimulation was therefore used as a task-design feature to strengthen functional coupling between cognitive control and motor execution, rather than as a therapeutic technique per se. Its integration within the CMDT protocol was aimed at enhancing anticipatory processing, inter-hemispheric coordination, and top-down control during movement, with operator-adjustable speed and intensity parameters.

### 2.8 Statistical analysis

Firstly, the normality of the distributions was assessed using the Shapiro–Wilk test, and the assumption of homogeneity of variance was examined using Levene’s test. For repeated-measures factors, the assumption of sphericity was evaluated using Mauchly’s Test of Sphericity, and when violated, Greenhouse–Geisser corrections were applied. The data were analyzed using mixed-design ANOVAs with Group (Experimental vs. Control) as the between-subjects factor and Time (T0, T1, T2) as the within-subjects factor. An additional 2-level within-subject factor was added for the STAI - the Anxiety Type (trait and state), and the 10mWT - Walking speed (comfortable and fast). Effect sizes were estimated using partial eta squared (η_p_^2^). Significant effects were followed by Bonferroni-corrected post hoc comparisons for unequal groups. Pearson’s correlational analyses were also performed between anxiety and depression levels and ERP components. The significance level was set at α = 0.05. All statistical analyses were performed using Statistica 12.0 (StatSoft Inc., Tulsa, OK, USA).

#### 2.8.1 Mediation analysis

To investigate whether changes in anticipatory neural activity mediated the effects of CMDT training on the expected emotional outcomes, a series of mediation analyses was conducted, focusing on anxiety and depression. Specifically, the mediation framework tested whether the effect of the intervention on changes in anxiety and depressive symptoms was transmitted through intervention-related changes in anticipatory ERP activity (pN and BP), thereby evaluating the potential mediating role of these neurophysiological markers. Change scores were computed for all relevant variables as the difference between post-intervention (T2) and baseline (T0) values. Group was set as the independent variable. The dependent variables were: (i) changes in anxiety, computed separately for state and trait anxiety, and (ii) depression. The mediators were changes in pN and BP components. Mediation analyses were performed using ordinary least squares regression with non-parametric bootstrapping (5,000 resamples) to estimate indirect effects and corresponding 95% bias-corrected confidence intervals. An indirect effect was considered statistically significant if the confidence interval did not include zero. All models included baseline values of the dependent variable as covariates to control for regression-to-the-mean effects. All analyses were conducted using standard mediation procedures of R (version 4.5.3). The significance level was set at α = 0.05.

## 3 Results

Shapiro–Wilk test results showed that at the group-specific level, most distributions did not significantly deviate from normality, with 42 out of 48 distributions meeting the Shapiro–Wilk criterion. Among the six distributions showing significant deviations, the magnitude of deviation was quantified using the Shapiro–Wilk W statistic and the complementary index 1–W. Three distributions showed marked deviations from normality, with W values ranging from 0.688 to 0.706 and 1–W values ranging from 0.294 to 0.312. These involved STAI-Y1 and BDI-II at T1 in the experimental group, and STAI-Y1 at T1 in the control group. The remaining three significant deviations were moderate, with W values ranging from 0.825 to 0.881 and 1–W values ranging from 0.119 to 0.175. These involved STAI-Y1 and BDI-II at T2. Overall, although some emotional measures showed departures from normality, most distributions satisfied the normality assumption. Given the overall distributional pattern and the robustness of mixed-design ANOVA to moderate deviations from normality, the planned parametric analyses were retained.

Mixed-design ANOVA results for emotional, motor, cognitive, and ERP measures are summarized in Table 1.

**TABLE 1:** Summary of mixed-design ANOVA results for emotional scores [anxiety (STAI), depression (BDI)], motor tests [balance (BBS), functional performance (TUG), and walking speed (10mMT)], cognitive outcomes [response time (RT), response time variability (ICV), and response accuracy (error rate)], and ERP outcomes [prefrontal Negativity (pN) and Bereitschaftspotential (BP)].

Domain	Outcome	Main effect Group (DoF = 1,28)	Main effect Time (DoF = 2,56)	Interaction Grou*p* × Time (DoF = 2,56)	*Post hoc* summary
Emotion	STAI	ns	F = 47.79, *p* < 0.01, ηp^2^ = 0.61	F = 27.21, *p* < 0.01, ηp^2^ = 0.50	EXP: T0 > T1**, T1 > T2** CTRL: ns
	BDI	F = 5.46, *p* < 0.05, ηp^2^ = 0.16	F = 31.97, *p* < 0.01, ηp^2^ = 0.53	F = 8.07, *p* < 0.01, ηp^2^ = 0.22	EXP: T0 > T1**, T1 > T2** CTRL: ns
Motor	BBS	ns	F = 10.94, *p* < 0.01, ηp^2^ = 0.28	ns	T0 < T1**, T0 < T2**
	TUG	ns	F = 3.39, *p* < 0.05, ηp^2^ = 0.11	ns	T0 > T2*, T1 > T2*
	10mWT	ns	F = 6.64, *p* < 0.01, ηp^2^ = 0.19	ns	T0 < T2**, T1 < T2*
Cognitive behavioral	RT	ns	F = 19.48, *p* < 0.01, ηp^2^ = 0.41	F = 23.68, *p* < 0.01, ηp^2^ = 0.46	EXP: T0 > T1**, T1 > T2** CTRL: ns
	ICV	ns	F = 14.66, *p* < 0.01, ηp^2^ = 0.34	F = 8.83, *p* < 0.01, ηp^2^ = 0.24	EXP: T0 > T1*, T1 > T2** CTRL: ns
	Error rate	ns	F = 14.70, *p* < 0.01, ηp^2^ = 0.34	F = 9.57, *p* < 0.01, ηp^2^ = 0.26	EXP: T0 > T1*, T1 > T2** CTRL: ns
Cognitive ERP	pN	F = 11.80, *p* < 0.01, ηp^2^ = 0.30	F = 80.78, *p* < 0.01, ηp^2^ = 0.74	F = 75.59, *p* < 0.01, ηp^2^ = 0.73	EXP: T0 < T1**, T1 < T2** CTRL: ns
	BP	F = 48.52, *p* < 0.01, ηp^2^ = 0.63	F = 83.14, *p* < 0.01, ηp^2^ = 0.74	F = 26.57, *p* < 0.01, ηp^2^ = 0.34	EXP: T0 < T1**, T1 < T2** CTRL: ns

Reported values include main effects of Group and Time, Group × Time interactions, and significant *post hoc* comparisons. DoF, Degree of Freedom; ns, non-significant.

*p < 0.05;

**p < 0.01.

### 3.1 Emotion tests

**FIGURE 4:**
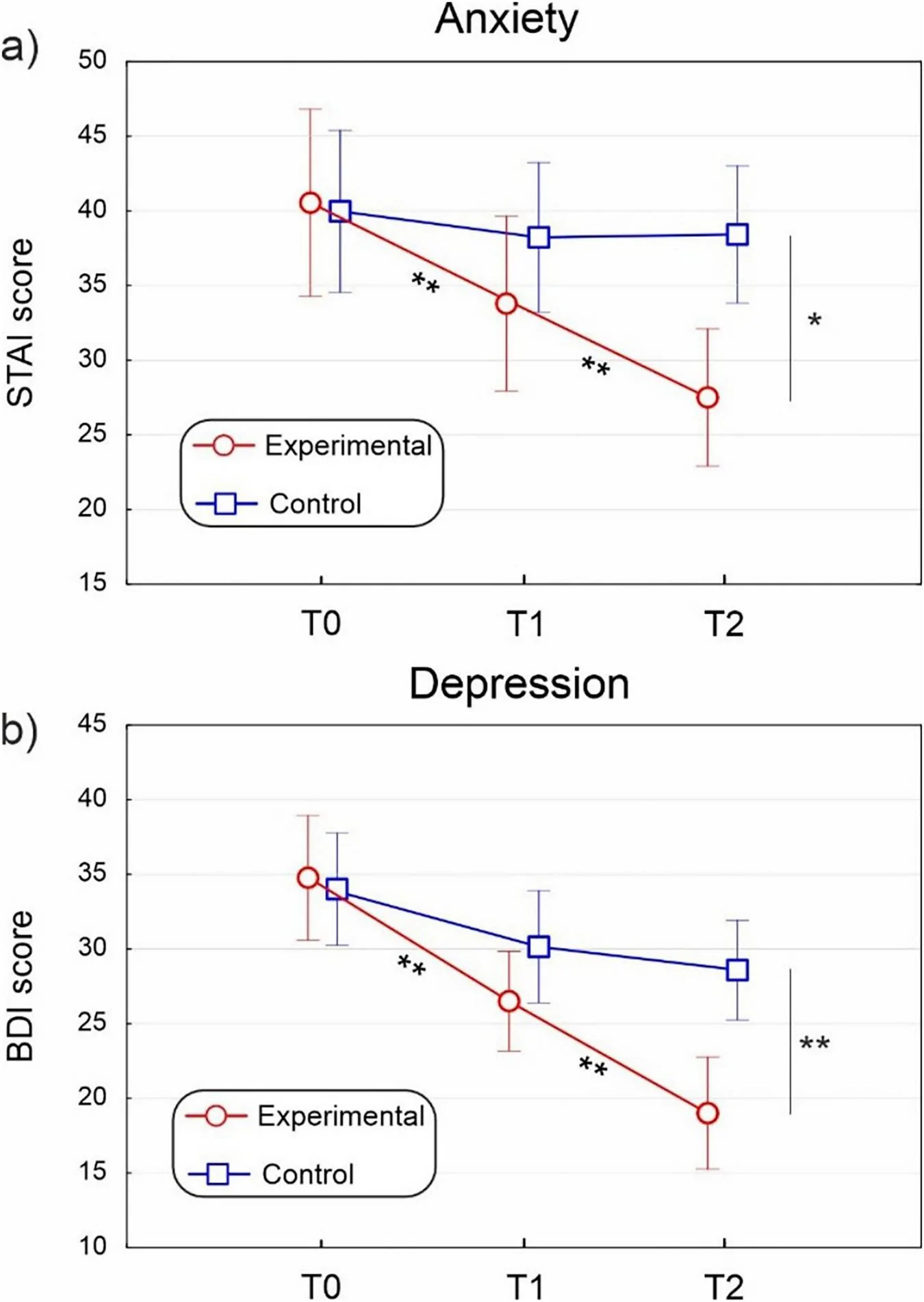
Interaction between Group and Time for: **(a)** anxiety (STAI) and **(b)** depression (BDI) scores. Vertical bars represent 95% confidence intervals. *p < 0.05, **p < 0.01.

The main effect of Time was significant for both STAI and BDI scores (all *p* < 0.01), whereas the main effect of Group was significant for BDI, but not for STAI (*p* = 0.17). For STAI, a significant main effect of Anxiety Type was also observed (*p* < 0.01), with higher trait (38.2 ± 4.3) than state (34.6 ± 4.9) anxiety scores. As reported in Figure 4, significant Group × Time interactions were observed for both anxiety and depression measures (all *p* < 0.01).

For STAI, post-hoc comparisons showed that, in the experimental group, STAI scores significantly decreased from T0 (40.5 ± 6.7) to T1 (33.9 ± 6.2) and further to T2 (27.5 ± 5.2). In contrast, the control group did not show significant changes over time, with no differences between T0 (39.9 ± 5.7), T1 (38.2 ± 5.4), and T2 (38.4 ± 4.9). In addition, while the anxiety level did not differ between groups at T0 and T1, at T2 the experimental group displayed lower anxiety (*p* < 0.05) than the control group. The other interactions were not significant (*p* > 0.10).

For BDI, *post hoc* comparisons showed that in the experimental group, scores significantly decreased from T0 (34.7 ± 5.1) to T1 (26.5 ± 4.4) and further to T2 (19.0 ± 2.6; *p* < 0.01). In the control group, no significant differences were observed across time (T0 = 33.8 ± 3.5, T1 = 30.1 ± 2.5, T2 = 28.6 ± 2.0). In addition, while the depression level did not differ between groups at T0 and T1, at T2 the experimental group displayed lower depression (*p* < 0.01) than the control group.

### 3.2 Motor tests

**FIGURE 5:**
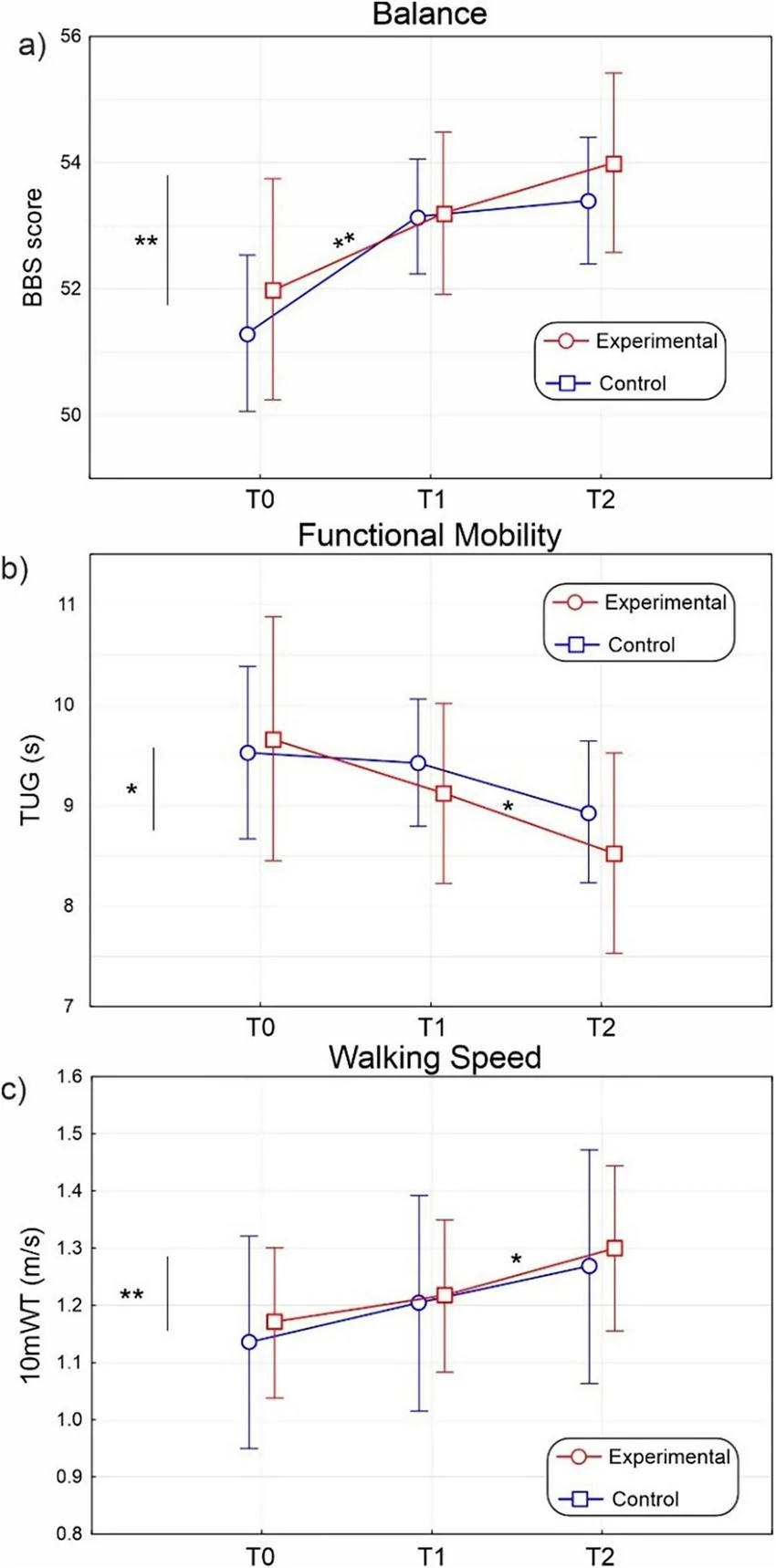
Interaction between Group and Time for: **(a)** balance (BBS), **(b)** functional performance (TUG), and walking speed (10mMT) tests. Vertical bars around the mean represent 95% confidence intervals. **(c)** Represents walking speed assessed using the 10-Meter Walking Test (10mWT). The lines on the left indicated the T0–T2 difference. Asterix refers to the main effect of Time. *p < 0.05, **p < 0.01.

For BBS, TUG, and 10mWT measures, no significant main effect of Group and Group × Time interactions were observed for any motor measure (Figure 5), whereas the main effect of Time was significant for all variables. Post-hoc comparisons revealed:

For the BBS, increased scores from T0 (51.7 ± 2.7) to T1 (53.2 ± 1.9; *p* < 0.01) and further from T0 to T2 (53.6 ± 2.2; *p* < 0.01).

For the TUG, a reduction of time to complete the exercise across time from T0 (9.6 s ± 1.8) to T2 (8.8 s ± 1.5; *p* < 0.05). The differences between T1 (9.5 s ± 1.4; *p* < 0.05) and T2 (*p* < 0.05), and between T0 and T2 were also significant (*p* < 0.01).

For the 10mWT, a speed increase across time from T0 (1.15 m/s ± 0.21) to T2 (1.29 s ± 0.24; *p* < 0.01). The differences between T1 (1.21 s ± 0.22; *p* < 0.05) and T2 (*p* < 0.05), and between T0 and T2 were also significant (*p* < 0.01). In addition, the main effect of Walking Speed was significant [F_(2,28)_ = 260.76, *p* < 0.01, η_p_^2^ = 0.90], with slower speed (*p* < 0.01) for comfortable (1.01 m/s ± 0.19) than fast (1.43 m/s ± 0.26) speed.

### 3.3 Cognitive test: behavioral results

**FIGURE 6:**
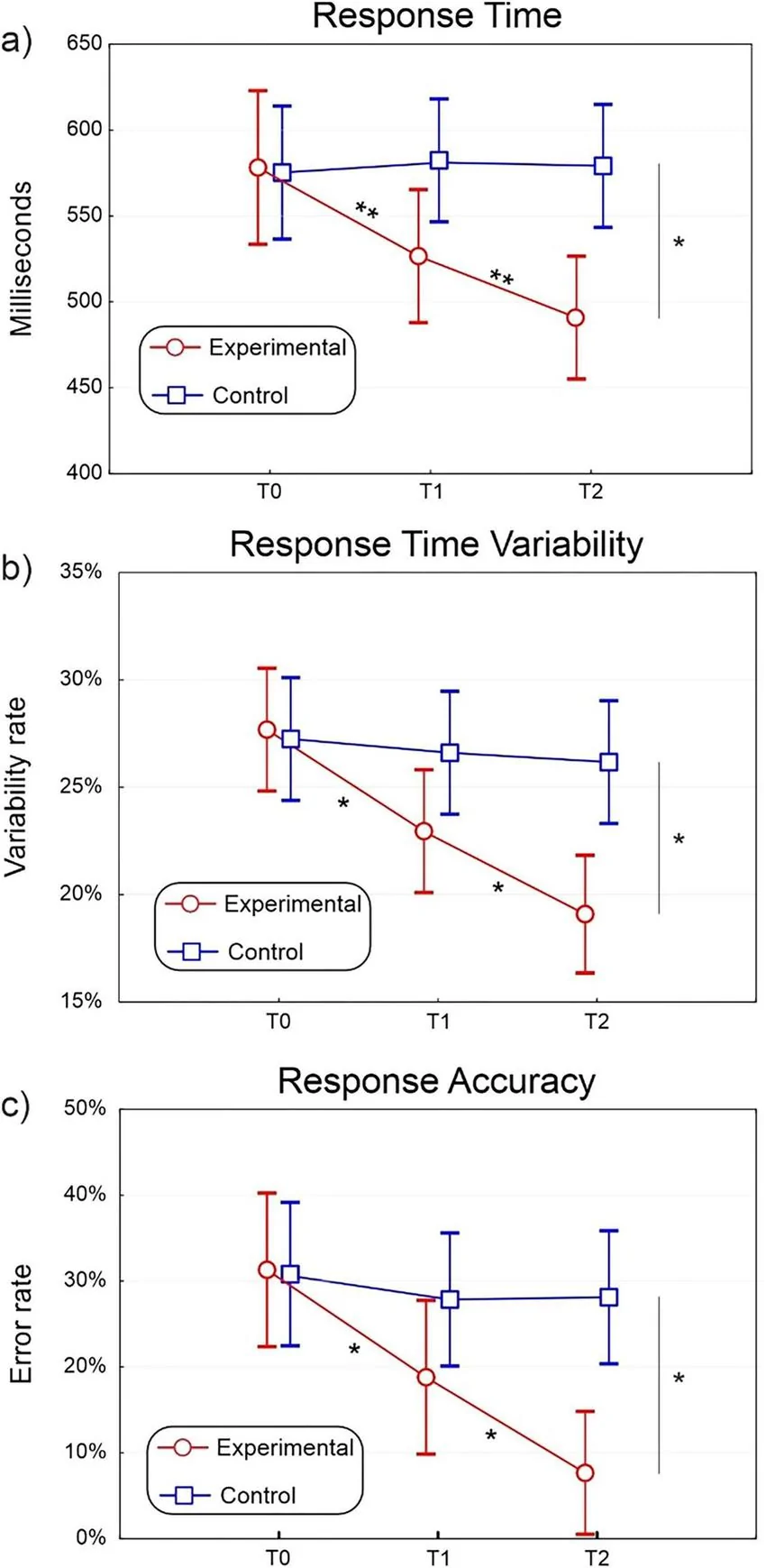
Interaction between Group and Time in the cognitive test. **(a)** Response time, **(b)** Response time variability, and **(c)** response accuracy (error rate). Vertical bars represent 95% confidence intervals. *p < 0.05, **p < 0.01.

For all measures (RT, RT variability, and error rate), the main effect of Group was not significant, whereas the main effect of Time was significant. However, significant Group × Time interactions were observed for all measures (all *p* < 0.01). *Post hoc* comparisons indicated in all cases a progressive reduction from T0 to T2 for the experimental group only, with no significant changes in the control group. At T2, the values of the experimental group were lower than those of the control group (*p* < 0.05). Data are shown in Figure 6.

The RT of the experimental group were: T0 = 578 ms ± 59, T1 = 527 ms ± 54, T2 = 490 ms ± 50. While in the control group were: T0 = 576 ms ± 57, T1 = 578 ms ± 58, T2 = 577 ms ± 59.

The RT variabilities of the experimental group were: T0 = 27.8% ± 3.3, T1 = 22.9% ± 3.8, T2 = 19.1% ± 3.4. In the control group were: T0 = 27.3% ± 2.5, T1 = 26.6% ± 2.8, T2 = 26.2% ± 2.6.

The error rates of the experimental group were: T0 = 31.3% ± 17.1, T1 = 18.8%, T2 = 7.7%. The control group error rates were: T0 = 30.6% ± 13.0, T1 = 27.9%, T2 = 28.1%.

### 3.4 Cognitive test: ERP results

**FIGURE 7:**
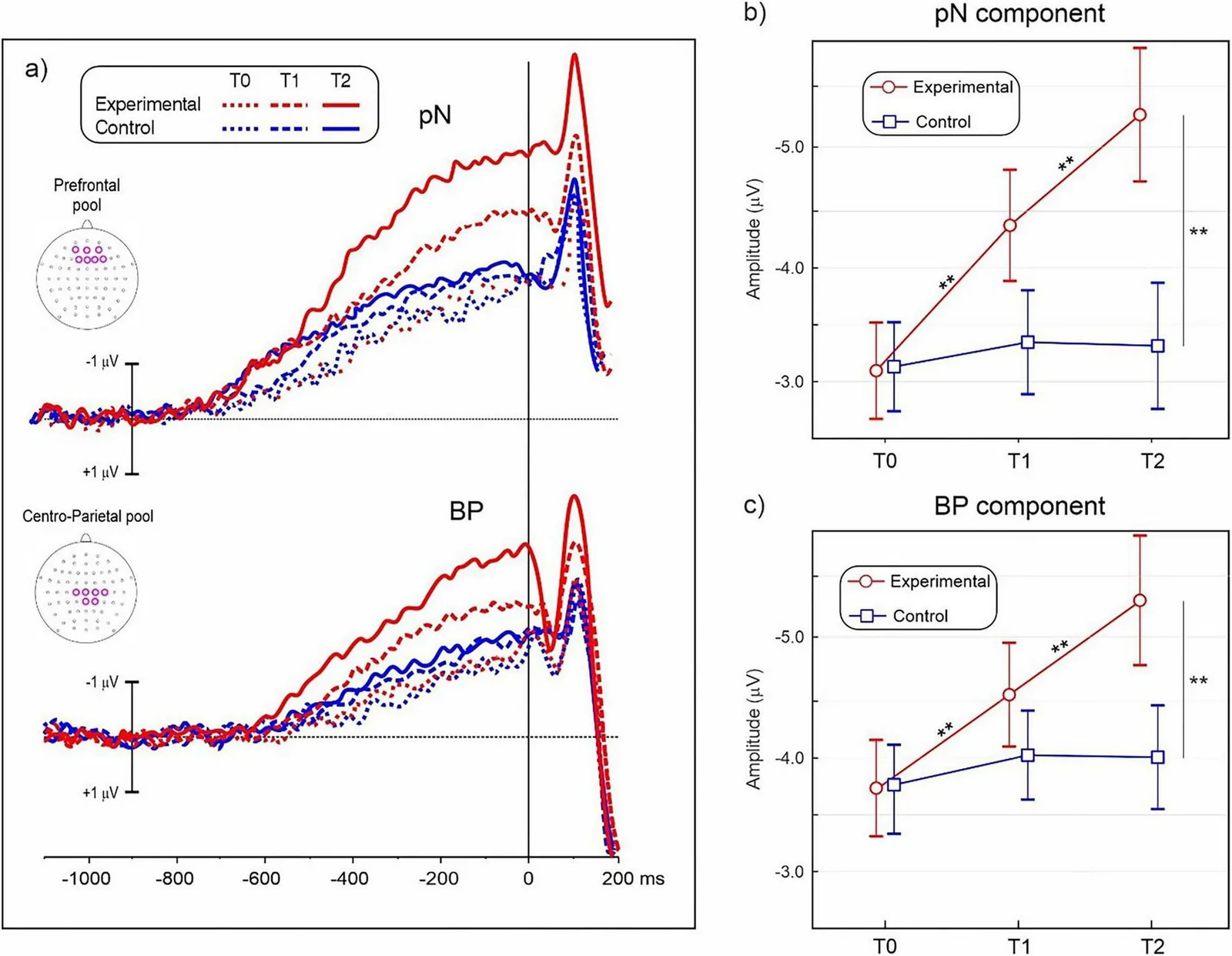
**(a)** Pre-stimulus ERP waveform for experimental and control groups across time (T0, T1, T2) over prefrontal (pN) and centro-parietal (BP) electrode pools. **(a)** Shows the prefrontal and the central pools of electrodes marked in Figure 3 and representing the pN and the BP, respectively. Amplitude changes in T0, T1, and T2 of the **(b)** pN and **(c)** BP components. Vertical bars represent 95% confidence intervals. **p < 0.01.

The average pre-stimulus ERP waveforms are represented in the two groups and conditions (Figure 7a). The BP component is detectable starting approximately from −600 ms before stimulus onset, emerging as a slow-rising negativity and reaching its peak at stimulus onset over medial central sites. The prefrontal negativity (pN) appears later, starting around −500 ms before stimulus onset and peaking immediately before stimulus presentation over medial prefrontal areas.

For both pN and BP amplitudes, significant main effects of Group and Time were observed. The Group × Time interaction for pN and BP amplitude was also significant and reported in Figures 7b, c, respectively. *Post hoc* comparisons showed progressive increases in anticipatory negativity from T0 to T2 in the experimental group, whereas no significant changes emerged in the control group.

### 3.5 Correlation results

**FIGURE 8:**
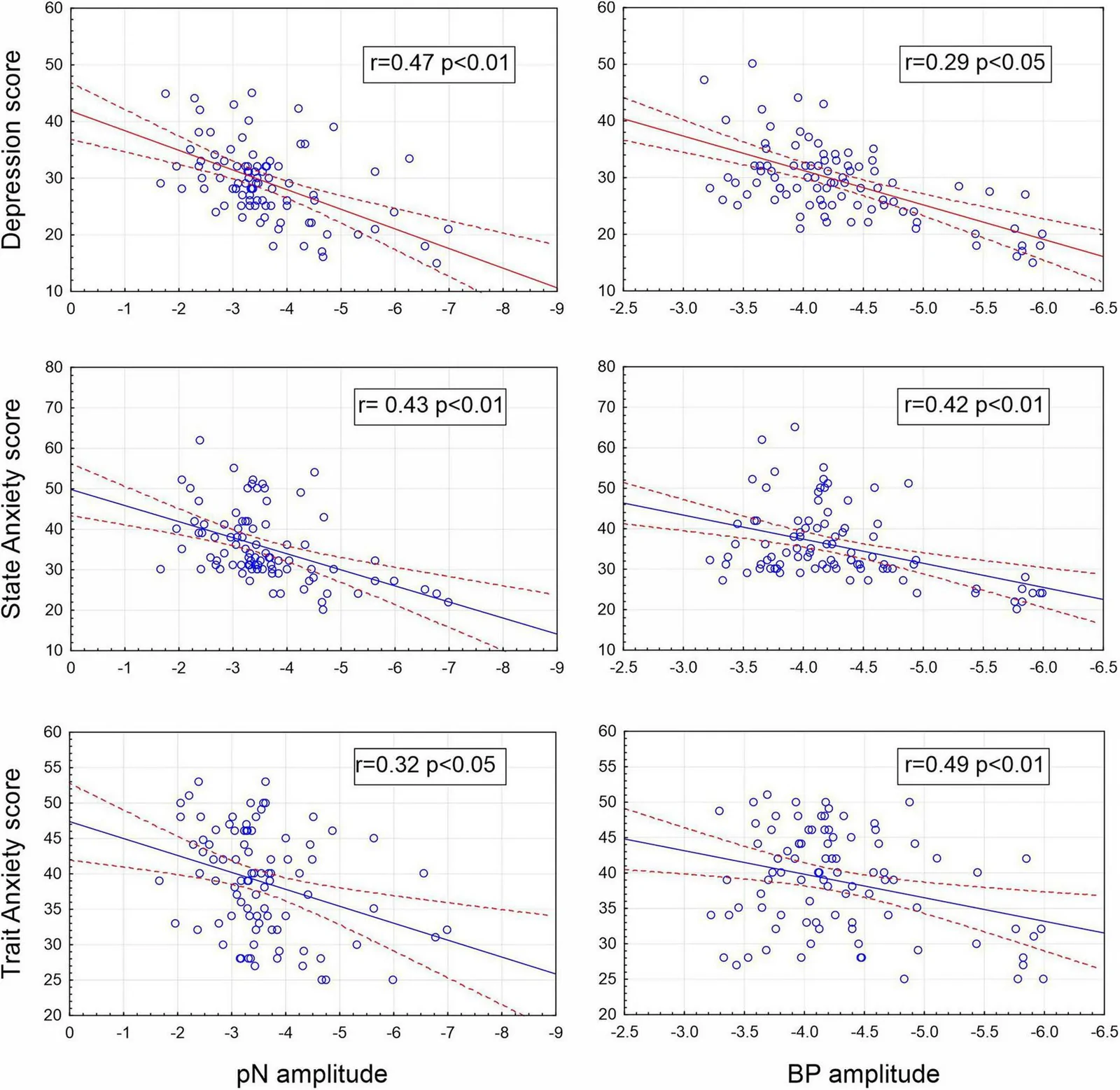
Scatter plots showing correlations between depression (BDI) or trait and state anxiety scores (STAI) and anticipatory pN and BP ERP components. Blue dots represent individual participants. Solid blue lines indicate linear regression fits, with dashed red lines representing 95% confidence intervals. Despite the positive r value, all correlations are negative, indicating that larger (more negative) anticipatory ERP amplitudes are associated with lower depression and anxiety levels.

Correlational analyses were conducted to examine the relationship between emotional measures and neurophysiological indices of anticipatory activity. As shown in Figure 8, significant negative correlations were observed between ERP amplitudes and both anxiety (STAI) and depression (BDI) scores, indicating that larger (i.e., more negative) anticipatory components were associated with lower symptom severity. Specifically, while both the pN and BP amplitudes show significant negative correlation with BDI and STAI scores, the pN had the strongest correlation with the depression (*r* = 0.47) and state anxiety (*r* = 0.43) scores, and the BP best correlated with the state (*r* = 0.42) and trait anxiety (*r* = −0.49) scores. Because pN and BP are negative-going ERP components, greater anticipatory activity is represented by more negative raw voltage values. Therefore, the negative regression slopes in Figure 8 indicate that larger pN/BP amplitudes are associated with lower emotional symptom scores

#### 3.5.1 Mediation analysis results

This analysis on anxiety scores revealed that changes in prefrontal anticipatory activity significantly mediated the effect of CMDT training on reductions in state anxiety. CMDT group assignment significantly predicted increases in pN amplitude (path a: β = −1.92, SE = 0.31, *p* < 0.01), indicating enhanced prefrontal anticipatory activity in the experimental group. In turn, greater increases in pN amplitude were associated with larger reductions in state anxiety after controlling for Group (path b: β = 0.41, SE = 0.14, *p* < 0.01). The indirect effect of CMDT training on state anxiety through the pN was statistically significant (ab = −0.79), with a 95% bootstrap confidence interval of −1.32, −0.28, indicating partial mediation. The direct effect of Group on state anxiety remained significant after inclusion of the mediator (c’: β = −1.48, SE = 0.52, *p* < 0.01), suggesting that CMDT exerted both direct and indirect effects on anxiety reduction. In contrast, the BP difference did not significantly mediate the effect of CMDT on state anxiety when entered as a mediator (indirect effect: ab = −0.18, 95% CI [−0.54, 0.12]). A similar pattern was observed for trait anxiety. The difference in the pN significantly mediated the effect of CMDT on trait anxiety reduction (indirect effect: ab = −0.63, 95% CI [−1.15, −0.21]), while the BP again failed to yield a significant indirect effect. These results indicate that prefrontal anticipatory control, rather than premotor preparation per se, plays a central role in CMDT-related anxiety reduction.

Mediation analyses conducted on changes in depressive symptoms revealed a weaker and more context-dependent mediation pattern. The CMDT group assignment significantly predicted increases in pN amplitude (path a identical to the anxiety models). Increased pN amplitude was associated with greater reductions in depressive symptoms (path b: β = 0.29, SE = 0.13, *p* < 0.05). The resulting indirect effect of CMDT on depression via the pN was significant but smaller in magnitude compared to anxiety (ab = −0.56, 95% CI [−1.07, −0.04]). However, once the pN was included in the model, the direct effect of Group on depression reduction was no longer significant (c’: β = −0.61, SE = 0.49, *p* = 0.22), indicating full mediation. This suggests that CMDT influenced depressive symptoms primarily through its effect on prefrontal anticipatory control, rather than through additional direct pathways. As observed for anxiety, the BP did not significantly mediate the effect of CMDT on depression (indirect effect: ab = −0.14, 95% CI [−0.46, 0.15]).

## 4 Discussion

The present study investigated the effects of a cognitive–motor dual-task training (CMDT) protocol incorporating structured alternating bilateral stimulation on emotional, cognitive, motor, and neurophysiological outcomes in frail and pre-frail older adults. The results provide converging behavioral and electrophysiological evidence that CMDT produces robust benefits across multiple domains, with particularly strong effects on anxiety regulation and executive efficiency, and more moderate but clinically relevant effects on depressive symptoms.

One of the central findings of this study is the marked enhancement of anticipatory brain activity indexed by pN and the BP following CMDT training. These ERP components are well-established markers of proactive cognitive control and motor preparation, respectively, and reflect the engagement of prefrontal–premotor networks before stimulus onset (Di Russo et al., 2019). The strong Group × Time interactions observed for both pN and BP amplitudes indicate that CMDT training leads to a progressive strengthening of anticipatory control processes, rather than merely reactive or post-stimulus processing. This pattern is consistent with neurocognitive models of aging that emphasize a shift from automatic to effortful control processes in older adults, particularly under demanding or dual-task conditions. CMDT appears to counteract age- and frailty-related reductions in preparatory control by repeatedly challenging attentional allocation, response selection, and inhibition within a motor context. Over time, this repeated engagement likely promotes functional reorganization and increased efficiency within frontal control networks, in line with compensatory and neuroplasticity frameworks of aging (e.g., Cabeza, 2002; Park and Reuter-Lorenz, 2009).

At the emotional level, CMDT training produced a pronounced reduction in both state and trait anxiety, with large effect sizes and a clear divergence from the motor-only control condition. Importantly, correlational analyses revealed that anxiety scores were strongly and negatively associated with both pN and BP amplitudes, indicating that individuals who exhibited greater enhancement of anticipatory neural activity also experienced larger reductions in anxiety. This finding provides mechanistic support for the interpretation that anxiety reduction following CMDT training is mediated by strengthened top-down anticipatory control. Anxiety is known to be closely linked to dysregulated prediction and excessive threat anticipation, often accompanied by impaired prefrontal modulation of subcortical and limbic structures (e.g., Williams, 2016; Tozzi et al., 2024). By reinforcing prefrontal engagement before action execution, CMDT may attenuate maladaptive anticipatory processes, leading to reduced hypervigilance and improved emotional regulation. Notably, the strongest correlations were observed between pN amplitude and anxiety measures, underscoring the pivotal role of prefrontal anticipatory activity in anxiety modulation. These results are consistent with prior work showing that enhanced proactive control and preparatory engagement can normalize anxiety-related deficits without the need for pharmacological or psychotherapeutic interventions (Lucia et al., 2024; Tuena et al., 2023; Yang et al., 2018).

Cognitive-Motor Dual-Task training produced more moderate reductions in depressive symptoms. Although depressive scores significantly decreased over time in the experimental group and differed from those of controls at the end of the intervention, effect sizes were smaller than those observed for anxiety, and interindividual variability was greater. This differential responsiveness aligns with contemporary transdiagnostic models, distinguishing anxiety and depression at the level of neural circuitry (e.g., Williams, 2016; Tozzi et al., 2024). While anxiety is strongly tied to anticipatory and proactive control mechanisms mediated by prefrontal networks, depression encompasses broader dysfunctions involving motivational, rewards-related, and affective systems, including reduced behavioral activation and diminished sensitivity to goal-directed outcomes (e.g., Admon and Pizzagalli, 2015; Kenwood et al., 2022).In the present study, self-reported depressive symptoms significantly decreased with CMDT. Additionally, mediation analyses revealed that increases in prefrontal anticipatory activity (pN) statistically explained this association, suggesting a possible role of anticipatory cognitive control in the observed improvement. Greater reductions in depressed symptoms were linked to higher increases in pN amplitude, which is consistent with this interpretation. These results, however, should be interpreted cautiously and do not permit definitive conclusions regarding whether CMDT influences depressive symptoms directly, indirectly through anticipatory control mechanisms, or through a combination of both pathways due to the relatively small sample size and the exclusive use of self-report measures. Taken together, these findings suggest that improvements in depressive symptoms may be associated with changes in anticipatory cognitive control. However, further studies involving larger samples, clinician-rated assessments, and longitudinal follow-up are needed to clarify the mechanisms underlying this association and to determine its generalizability. The heterogeneous findings reported in previous studies may also reflect differences in participant characteristics, such as frailty status and executive functioning, as well as methodological variability across studies, including the use of different self-report questionnaires, clinician-rated scales, and assessment procedures to evaluate depressive symptoms. (e.g., Nascimento et al., 2022; Schoene et al., 2014, 2015; Ye et al., 2024).

Mediation analyses clarified the mechanisms through which CMDT training influenced emotional outcomes. Changes in prefrontal anticipatory activity (pN) significantly mediated the effects of CMDT on both anxiety and depressive symptoms, but with distinct mediation patterns. For anxiety, pN partially mediated the CMDT effect, indicating that anxiety reduction involved enhanced anticipatory prefrontal control alongside additional training-related mechanisms. This is consistent with models linking anxiety to dysregulated anticipatory processing and impaired top-down control. In contrast, the effects of CMDT training on depression were fully mediated by changes in pN amplitude. Once anticipatory prefrontal activity was accounted for, the direct effect of CMDT on depressive symptoms was no longer significant, suggesting that depression reduction occurred indirectly through restoration of goal-directed control rather than direct mood modulation. This finding provides a mechanistic explanation for the smaller and more variable antidepressant effects of CMDT observed in the literature (e.g., Nascimento et al., 2022; Schoene et al., 2014, 2015; Ye et al., 2024). Premotor preparatory activity (BP) did not significantly mediate emotional outcomes, underscoring the central role of cognitive (rather than purely motor) anticipatory processes in emotional regulation. Overall, these findings indicate that CMDT exerts stronger and more robust effects on anxiety, while its influence on depression is more context-dependent and relies specifically on restoring anticipatory prefrontal control (e.g., Boccacci et al., 2026).

An important and innovative aspect of the present CMDT protocol lies in the integration of alternating bilateral stimulation as a task-design feature rather than as a psychotherapeutic technique per se. Bilateral stimulation was embedded within cognitive–motor exercises to continuously challenge lateralized stimulus–response mapping, interhemispheric coordination, and attentional shifting. From a neurophysiological perspective, this design likely amplified engagement of frontal control systems by preventing automatization and enforcing sustained anticipatory processing. Previous evidence indicates that bilateral stimulation modulates interhemispheric coherence and prefrontal–limbic connectivity, supporting the integration of cognitive and emotional information (Christman et al., 2003; Propper et al., 2007; Pagani et al., 2012, 2015; Herkt et al., 2014; Stingl et al., 2025). In the present study, the progressive modulation of stimulation speed and complexity may have contributed to the observed escalation of pN and BP amplitudes, thereby enhancing the regulatory impact of CMDT on emotional outcomes. Although the present design does not allow isolation of the specific contribution of bilateral stimulation relative to CMDT alone, the magnitude of the anticipatory and emotional effects suggests that its inclusion may potentiate control-related mechanisms, particularly in frail individuals with reduced cognitive reserve. However, these mechanisms were not directly evaluated in the present study and are discussed solely in light of previous evidence.

Beyond emotional and neurophysiological outcomes, CMDT improved executive efficiency during the cognitive task, as reflected by faster response times, reduced variability, and improved accuracy, alongside generalized gains in balance, mobility, and gait speed observed in both groups. These findings indicate that CMDT does not compromise physical function and may even facilitate safer and more efficient motor behavior under cognitive load, an ability that is critical for daily functioning and fall prevention in frail older adults (Woollacott and Shumway-Cook, 2002). From a translational perspective, the present results highlight CMDT as a low-cost, non-pharmacological intervention capable of addressing multiple dimensions of frailty, including emotional vulnerability and cognitive decline. The strong effect on anxiety, combined with context-dependent benefits for depression, positions CMDT as a promising component of personalized, mechanism-based rehabilitation programs for aging populations (Clegg et al., 2013; Boccacci et al., 2026).

Some limitations should be acknowledged. First, the sample size was relatively small, and the merging of the two motor-only groups, although methodologically justified, may limit fine-grained comparisons across different types of physical training. Second, the absence of follow-up assessments precludes conclusions regarding the long-term persistence of training effects (Clegg et al., 2013; Rockwood and Mitnitski, 2007). Third, only self-report questionnaires (BDI-II and STAI-Y) were used to measure emotional outcomes. Despite their widespread use and strong validation, these tools are prone to response biases and do not distinguish between subclinical symptomatology and clinically recognized diseases. To give a more thorough assessment of emotional functioning, future research should use clinician-administered tests or organized diagnostic interviews. Fourth, while correlational analyses suggest mechanistic links between anticipatory brain activity and emotional outcomes, causal mediation analyses were not performed. Future studies should examine the durability of CMDT training effects, explore dose–response relationships, and disentangle the specific contribution of bilateral stimulation (Tuena et al., 2023; Wollesen et al., 2020). Combining CMDT with neuroimaging or neuromodulation techniques may further clarify the neural pathways underlying its emotional and cognitive benefits and may enhance its antidepressant efficacy. Although exercise intensity was standardized through the training protocol, no objective physiological measures (e.g., heart rate or ratings of perceived exertion) were collected to quantify the internal training load. Future studies should incorporate these measures to better characterize the relationship between exercise intensity and neurophysiological outcomes.

From a translational perspective, the principles underlying CMDT may be effectively implemented in community and clinical settings through the integration of interactive technological devices, such as Witty SEM systems, combined with structured motor exercises and cognitive stimulation. These tools allow the delivery of dual-task activities that simultaneously engage motor performance and executive functions in a dynamic and adaptable environment. For example, participants may perform walking or stepping tasks in response to visual or auditory stimuli generated by the device, requiring rapid decision-making, inhibition control, or rule-switching. Balance and coordination exercises can be paired with tasks involving selective attention, working memory, or stimulus discrimination, such as responding only to specific colors or sequences. Additionally, strength or mobility exercises may incorporate cognitive challenges like pattern recognition or reaction-time tasks, enhancing both physical and cognitive engagement. These technology-assisted CMDT approaches offer a scalable, engaging, and customizable strategy for promoting cognitive, motor, and emotional health in older adults, although further studies are needed to confirm their effectiveness in real-world implementation contexts.

In conclusion, the present study demonstrates that CMDT training incorporating alternating bilateral stimulation produces robust improvements in anticipatory neural control, executive efficiency, and emotional regulation in frail older adults. CMDT exerts particularly strong effects on anxiety by reinforcing prefrontal anticipatory mechanisms, while its impact on depression is more moderate, context-dependent, and mediated by restored goal-directed control. These findings support CMDT as a mechanism-driven intervention that targets core control processes underlying emotional vulnerability in aging, offering a valuable and scalable approach for promoting mental and functional health in frail populations (Clegg et al., 2013; Bartoli et al., 2020; Pozo et al., 2023). Beyond its clinical implications, the present findings contribute to a reconceptualization of frailty as a condition marked by impaired anticipatory control in addition to reduced physical capacity (Rockwood and Mitnitski, 2007; Boccacci et al., 2026). The strong coupling between prefrontal anticipatory activity and emotional outcomes observed here suggests that frailty involves a diminished ability to proactively regulate action and emotion before external demands arise. CMDT appears to counteract this deficit by restoring control processes upstream of behavior, offering a mechanistic framework for understanding how targeted cognitive–motor interventions can improve emotional resilience in vulnerable aging populations (Cabeza, 2002; Park and Reuter-Lorenz, 2009).

## Data Availability

The raw data supporting the conclusions of this article will be made available by the authors, without undue reservation.
