# Supplementary material for: Restoring anticipatory control through cognitive–motor dual-task training improves emotional and executive function in frail older adults
Source: Front Aging Neurosci. 2026 Aug 14;18:1890281. doi: 10.3389/fnagi.2026.1890281 (PMC13523101; doi:10.3389/fnagi.2026.1890281)
Supplement: Supplementary file 1 [file Supplementary_file_1.docx]

**Supplementary Material**

**Disclosure of AI-Assisted Generation of Figure 1**

**Article title:**
*Restoring Anticipatory Control Through Cognitive–Motor Dual-Task Training Improves Emotional and Executive Function in Frail Older Adults*

**AI Tool**

- Tool: Microsoft 365 Copilot
- Provider: Microsoft Corporation
- Access: Institutional Microsoft 365 license provided by the University of Rome Foro Italico
- Purpose: Generation of an image for Figure 1

**Prompt(s)**

*Initial prompt*

Create an original full-body side-view photo of a sporty senior man with grey hair, performing a goblet squat holding a black kettlebell, isolated on a clean white background, studio lighting, professional medical publication style.

*Subsequent refinement*

Recreate the image as a drawing/illustration rather than a photograph.

**Extent of AI Use**

The image for Figure 1 was generated using Microsoft 365 Copilot on the basis of the prompts reported above.

No external image files, photographs, drawings, screenshots, or third-party copyrighted images were uploaded to the system during the generation process.

The authors selected one of the generated outputs and used it as an illustrative representation of the goblet squat exercise described in the manuscript.

**Attribution**

Figure 1 was generated using Microsoft 365 Copilot from text prompts provided by the authors.
